# Machine Learning-Based Diagnosis and Ranking of Risk Factors for Diabetic Retinopathy in Population-Based Studies from South India

**DOI:** 10.3390/diagnostics13122084

**Published:** 2023-06-16

**Authors:** Abhishek Vyas, Sundaresan Raman, Sagnik Sen, Kim Ramasamy, Ramachandran Rajalakshmi, Viswanathan Mohan, Rajiv Raman

**Affiliations:** 1 Birla Institute of Technology & Science, Pilani 333031, India; 2 Aravind Eye Hospital, Madurai 625020, India; 3 Moorfields Eye Hospital, London EC1V 2PD, UK; 4 Dr. Mohans Diabetes Specialities Centre, Chennai 600086, India; 5 Shri Bhagwan Mahavir Vitreoretinal Services, Sankara Nethralaya, Chennai 600006, India

**Keywords:** diabetic retinopathy, ranking, risk factors, machine learning

## Abstract

This paper discusses the importance of investigating DR using machine learning and a computational method to rank DR risk factors by importance using different machine learning models. The dataset was collected from four large population-based studies conducted in India between 2001 and 2010 on the prevalence of DR and its risk factors. We deployed different machine learning models on the dataset to rank the importance of the variables (risk factors). The study uses a *t*-test and Shapely additive explanations (SHAP) to rank the risk factors. Then, it uses five machine learning models (K-Nearest Neighbor, Decision Tree, Support Vector Machines, Logistic Regression, and Naive Bayes) to identify the unimportant risk factors based on the area under the curve criterion to predict DR. To determine the overall significance of risk variables, a weighted average of each classifier’s importance is used. The ranking of risk variables is provided to machine learning models. To construct a model for DR prediction, the combination of risk factors with the highest AUC is chosen. The results show that the risk factors glycosylated hemoglobin and systolic blood pressure were present in the top three risk factors for DR in all five machine learning models when the *t*-test was used for ranking. Furthermore, the risk factors, namely, systolic blood pressure and history of hypertension, were present in the top five risk factors for DR in all the machine learning models when SHAP was used for ranking. Finally, when an ensemble of the five machine learning models was employed, independently with both the *t*-test and SHAP, systolic blood pressure and diabetes mellitus duration were present in the top four risk factors for diabetic retinopathy. Decision Tree and K-Nearest Neighbor resulted in the highest AUCs of 0.79 (*t*-test) and 0.77 (SHAP). Moreover, K-Nearest Neighbor predicted DR with 82.6% (*t*-test) and 78.3% (SHAP) accuracy.

## 1. Introduction

Diabetes mellitus (DM) is a metabolic syndrome with an increasing prevalence and high mortality rate [1]. The prevalence of diabetes in people aged 20–79 years has increased from 61.3 million in 2011 to 77 million today, and a further 77 million are pre-diabetic, raising significant concerns about the public health burden of this condition [2,3]. By 2030, it is estimated that approximately 101 million people in India will have diabetes [4,5,6].

Diabetic retinopathy (DR) is a common ocular complication of DM and is considered one of the leading causes of vision loss and impairment in adults in the working-age group [7,8]. According to a cross-sectional survey in England in 1990–1, the leading cause of blindness was macular degeneration, which accounted for 49% of blind registrations, and glaucoma was at 12%, and diabetes was at 4%. However, in the working-age group of 16–64 years, diabetic retinopathy was attributed to 12% of blindness, while diabetes was the most critical cause of blindness [9].

The number of people affected by diabetes-related retinal disease is 382 million worldwide, and by 2025, that number is anticipated to rise to 592 million [10]. The estimated prevalence of DR is around 34.6% (approximately 93 million individuals), and 10.2% have an advanced stage of the disease [11]. According to the National Diabetes and Diabetic Retinopathy Survey report 2015–2019, India has a DR prevalence of 11.8% in the population aged above 50, and 10.6% of patients are at risk of losing vision [12].

As DR typically does not manifest symptoms until the disease has progressed, only screening methods, such as a routine eye exam or retinal photography, can detect the disease in its early stages. However, because of the increasing number of people diagnosed with diabetes, systematic screening of all people with diabetes may be a big challenge.

DR is also predicted in the literature using deep learning systems. Developing deep learning systems requires standardized grading of retinal images, which is often a challenge. Though screening through retinal photographs is the gold standard, it is important to identify the groups with the highest risk of developing DR so that photographic screening can be prioritized, especially in populations with a high prevalence of diabetes. Therefore, there is a need to look for systemic factors related to DR, which can play a key role as a prescreening tool for DR.

Several factors, such as high blood pressure, postprandial hyperglycemia, albuminuria, serum creatinine, glycosylated hemoglobin, and plasma glucose levels, are significantly associated with the risk of DR [13,14,15,16,17,18,19]. Therefore, understanding the role of risk factors is important for developing a strategy to improve global eye health. A previous study has determined that diabetes patients older than 50 years with diabetes duration > 5 years and systolic blood pressure > 140 mm Hg could be targeted to achieve optimal detection of vision-threatening diabetic retinopathy [20].

Risk factors for DR have been identified using statistical techniques in the literature [21,22,23,24,25,26,27]. Moreover, the ranking of various risk factors for DR has not received much attention in the literature. Ranking risk factors aims to streamline screening programs and focus on the most important ones.

Clinical data on risk factors has been used in the literature to predict DR. In this context, Cichosz et al. [28] used a linear classification model to predict which individuals had diabetic retinopathy based on data obtained from the National Health and Nutritional Examination Survey (NHANES, 2005–2008) [29] on the oral glucose tolerance test (OGTT), FPG, or HbA1C, and retinal imaging. Using information regarding HbA1c, BMI, waist circumference, age, SBP, urinary albumin, and urinary creatinine, they constructed a model that predicts the presence of retinopathy with a negative predictive value of 99% and a positive predictive value of 22%. Ogunyemi et al. [30] used clinical data from urban safety-net clinics and public health data from the Centers for Disease Control and Prevention (CDC) National Health and Nutrition Examination Survey to learn RUSBoost [31] and AdaBoost [32] ensemble classifiers for predicting retinopathy. The results show that the clinical dataset was not very good at predicting diabetic retinopathy. The best RUSBoost ensemble had an accuracy of 73.5%, a sensitivity of 69.2%, a specificity of 55.9%, and an AUC of 0.72 on cases that had never been seen before (the test data). Tsao et al. [33] built a prediction model for the DR in type 2 diabetes mellitus using data mining techniques, including Decision Trees, Support Vector Machines, Logistic Regressions, and Artificial Neural Networks. The performance of Support Vector Machines was better than that of the other machine learning algorithms. It achieved an accuracy of 79.5% and an AUC of 0.839 using a percentage split (i.e., the data set was divided into 80% as training and 20% as a test).

As risk factors have been used to predict DR, it is important to screen significant risk factors from the many presented in the dataset. There is no method to identify the top risk factors for DR. The paper gives a novel approach to ranking risk factors to identify the most significant ones. These risk factors could aid in developing a risk factor-based algorithm that can aid in the prescreening of DR. The algorithm can help as a prescreening tool for detecting the need for using fundus photographs for identifying referable and non-referable DR.

With a myriad of risk factors for most diseases, it is essential to identify the most important ones, which can be fed to machine learning models for improved classification. We have developed an algorithm for ranking the risk factors for diabetic retinopathy. We have incorporated two techniques for ranking: first, statistical methods (using *p*-values) and second, Shapley Additive Explanations (SHAP). Each of these two methods serves as a validation for the other. Our proposed algorithm can potentially rank risk factors for any other disease.

Our study also predicts DR using five machine-learning classification models, Decision Tree (DT), Support Vector Machines (SVM), K-Nearest Neighbors (KNN), Logistic Regression (LR), and Naive Bayes (NB). Finally, we have employed an ensemble of machine learning models to predict DR.

The paper is organized as follows: Section 2 discusses materials and methods. Section 3 gives the results, followed by a Discussion in Section 4 and a Conclusion in Section 5.

## 2. Material and Methods

### 2.1. Samples and Data Preprocessing

The sample dataset was collected from four large population-based studies conducted in India between 2001 and 2010 on the prevalence of DR and its risk factors [34,35,36,37]. All methods were performed following the relevant guidelines and regulations. The study was approved by the Institutional Review Boards of Madras Diabetic Research Foundation, Chennai, India; Vision Research Foundation, Chennai, India; and Aravind Eye Care System, Madurai, India. Informed consent was obtained from the participants according to the Declaration of Helsinki before collecting the data. These studies had patient-level data and included previously diagnosed and newly diagnosed diabetics. In this study, we included data on people aged 40 and older to obtain uniform data for analysis. In the current study, the diagnosis of new diabetes was defined as FBS > 7 mmol/L or >126 mg/dL at the time of initial screening. Age at presentation, duration of diabetes (for known individuals with diabetes), gender, history of hypertension, obesity, cardiovascular disease (CVD), and smoking history were among the sociodemographic and clinical parameters shared by all studies. The prevalence of the stages of DR is 1% proliferative DR, 12.4% mild/moderate non-proliferative DR, 1.4% severe non-proliferative DR, and 3.7% diabetic macular edema.

Data from all four studies was entered into a Microsoft Excel spreadsheet. The total number of people with DR was 857, and those without DR were 3133. An ophthalmologist provided a group of features contributing to the disease directly and indirectly. We call these features risk factors, and our primary objective in this study is to rank these risk factors, which include the history of hypertension status, insulin treatment status, systolic blood pressure status, glycosylated hemoglobin (HBA1c) value, duration of diabetes mellitus, fasting blood glucose, gender, body mass index, and age. Ordinal encoding is applied to categorical risk factors to convert them into continuous risk factors as machine learning models only understand numbers in data. Normalization was applied to continuous risk factors so that the deviation of the variables did not affect classification or model interpretation. Like previous medical data studies, we replaced the missing values with the mode for binary data and the median for numerical data [38].

Ranking features can be done using Random Forests and Logistic Regression. If Random Forests are used, equal importance is given to correlated features. Furthermore, they give preference to features with high cardinality. Logistic regression assumes linearity between the dependent variable and the independent variables. Moreover, it requires no multicollinearity between independent variables. An independent two-sample *t*-test ranks the risk factors according to their *p* values. A lower *p*-value denotes more importance. Furthermore, Shapely additive explanations [39] are used, giving Shapely values a suitable measure of feature importance. The higher the Shapely value, the higher is the importance of the feature. The coding was performed in Python using a Google Colaboratory notebook with a CPU frequency of 2.30 GHz, 2 CPU cores, the Haswell CPU family, and 12 GB of available RAM. The Python libraries used were sklearn, imblearn, numpy, pandas, matplotlib, collections, and scipy. The purpose of using machine learning classification models is to validate the *t*-test and SHAP-based rankings.

### 2.2. t-Test

A *t*-test (also known as the student’s *t*-test) is a tool for evaluating the means of one or two populations using hypothesis testing. A *t*-test may be used to evaluate whether a single group differs from a known value (a one-sample *t*-test), whether two groups differ from each other (an independent two-sample *t*-test), or whether there is a significant difference in paired measurements (a paired or dependent samples *t*-test). An independent two-sample *t*-test ranks the risk factors according to their *p* values. A lower *p*-value denotes more importance.

The *t*-test assumes that the independent samples of two populations have the same variance and are normally distributed. As there are two samples from a population with unequal variances, the *t*-test is reasonably robust to the violation of its first assumption. A *t*-test repeated measure design yields small effects due to the small sample error. It also results in the effective management of individual differences. One group is available for testing, which may result in less data noise.

### 2.3. Shapley Values

Lloyd Shapely, in 1953 [40], proposed the concept of Shapley values, which numerically evaluate the value of playing a game. It is important to interpret a model’s prediction correctly. It provides an insight into how a model may be improved, engenders user trust, and supports understanding the modeled process. The model itself is the best explanation of a simple model. A simple explanation model is used for complex models, such as ensembles or deep networks, as an interpretable approximation of the original model. In multicollinearity, Shapley regression values are feature importances for linear models. The method requires the model to be retrained on all feature subsets S ⊆ F, where F is the set of all features. It assigns to each feature an importance value that represents the effect on the model prediction, including that feature. To compute this effect, a model f_S∪{i}_ is trained with that feature present, and another model f_S_ is trained with the feature withheld. Then, from the two models, predictions are compared with the current input f_S∪{i}_(x_S∪{i}_) − f_S_(x_S_), where x_S_ represents the values of the input features in the set S. The preceding differences are computed for all possible subsets S ⊆ F\{i} because the effect of withholding a feature depends on other features in the model. The Shapley values are then computed and used as feature attributions. They are a weighted average of all possible differences:

(1) $$\varphi_{i}=\Sigma \frac{\;\left|S\right|!\left(\left|F\right|-\left|S\right|-1\right)!}{\left|F\right|!\;}\;\;\;\;[f_{S\cup \{i\}}(x_{S\cup \{i\}})-f_{S}(x_{S})]$$

S ⊆ F\{i}

Shapely values are a unified measure of feature importance. These are solutions to Equation (1) as they are the Shapley values of a conditional expectation function of the original model. The higher the Shapely value, the higher is the importance of the feature.

SHAP is used because it has a solid theoretical foundation in game theory. Furthermore, among the feature values, the prediction is fairly distributed. Moreover, SHAP has a fast implementation for tree-based models. Although Shapley value computation requires exponential time complexity, machine learning applications employ Shapley value approximation methods, such as Monte Carlo permutation sampling, which approximates Shapley value in linear time [41,42,43].

### 2.4. Method Design

The data was divided into an 80% training and 20% test set. Synthetic Minority Oversampling Technique (SMOTE) [44] was applied to the training data as the oversampling method to generate synthetic data in the minority class for solving the class imbalance problem. Following this, the proposed algorithm (Algorithm 1), as shown below, was applied to generate the weights for the risk factors using a *t*-test and SHAP independently.
**Algorithm 1:** Ranking of Risk Factors.**Begin****Input:** Set of risk factors (R = R_1_, R_2_, R_3_…, R_N_)1. **for** each risk factor, R_i_ **do**2.  Apply independent sample *t*-test to R_i_ and calculate its *p*-value. A lower *p*-value denotes more importance.3.  Apply SHAP to each model, namely, SVM, DT, KNN, LR, and NB, to calculate the Shapley value of R_i_ for each model. A higher Shapely value denotes higher importance.4.  **end for**5. Sort the risk factors in step 2 in increasing order of their *p*-values.6. Sort the risk factors in step 3 in decreasing order of Shapely values.7. The sorted risk factors are the risk factors R_1_, R_2_, R_3_…, R_N_ ranked independently using a *t*-test and SHAP.8. The weights of risk factors R_1_, R_2_, R_3_…, R_N_ are set based on the ranking results. For a  risk factor R_i_ with rank order *r*, its weight is set as *d* − *r*, where *d* is the number of   risk factors.9. Set the previous cross_val_score as prev_AUC = 010. **for** each i in the *N* (number of risk factors) ranked with the *t*-test and SHAP **do**11.  **for** each model j in M(SVM, DT, KNN, LR, and NB) **do**12.   Calculate the cross_val_score for the risk factor i as curr_AUC13.   **if** prev_AUC ≥ curr_AUC14.      Remove the risk factor from the ranked order and set its weight to         zero.15.   **else**  prev_AUC = curr_AUC16.  **end for**17. **end for**18. **return** Weights assigned to Risk factors for all models in the *t*-test and SHAP.**Output:** Weights assigned to risk factors for all models in the *t*-test and SHAP.**End**

**Figure 1:**
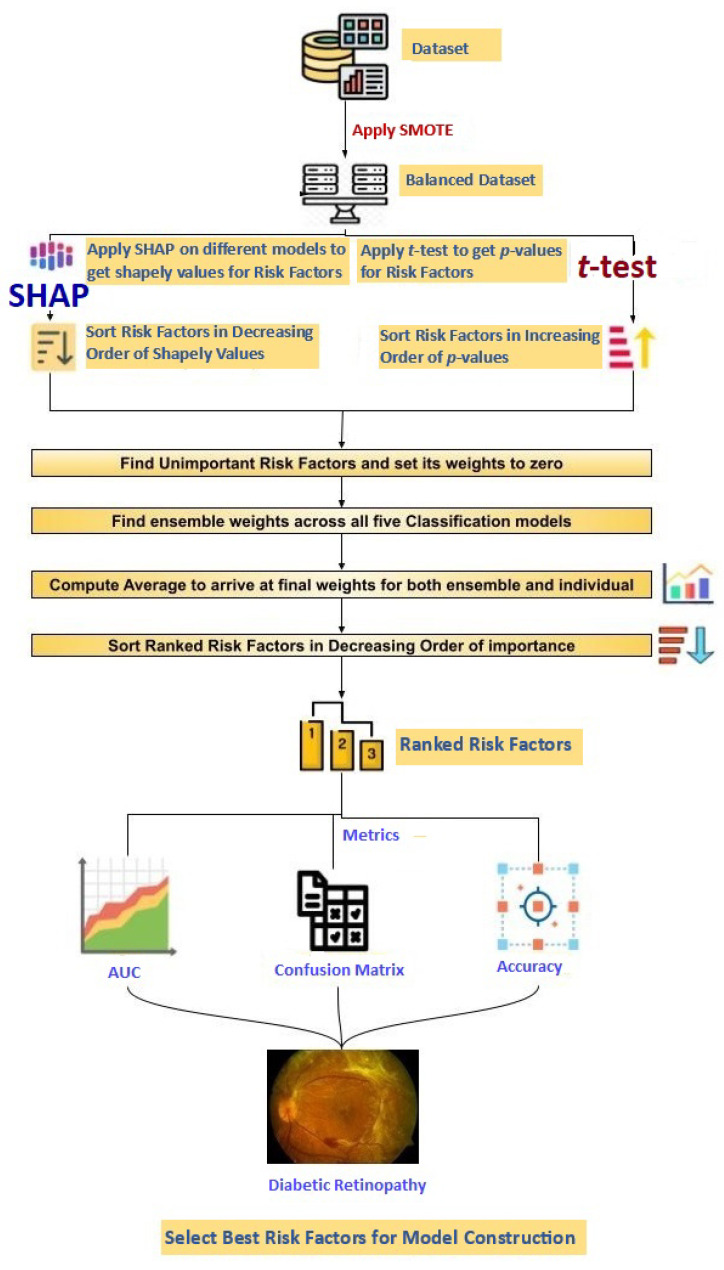
The flow of the method design.

The ensemble weights across all five models, namely, K-Nearest Neighbor (KNN) [45], Decision Tree (DT) [46], Support Vector Machine (SVM) [47], Logistic Regression [48], and Naive Bayes [49] are computed for both *t*-test and SHAP. The above steps are repeated three times to enhance robustness. An average is computed to arrive at the final model weights (for both the individual model and the ensemble case). As can be seen from Figure 1, the weights are sorted in decreasing order of importance for each model in the *t*-test and SHAP to generate the ranking of risk factors. The ranked risk factors are added individually to compute the best combination of risk factors producing the highest AUC metric to predict DR separately using a *t*-test and SHAP.

The *t*-test is used because it is robust to the violation of its assumption that the independent samples of two populations have the same variance and are typically distributed. SHAP is used in the algorithm because it has a solid theoretical foundation and fairly distributes the prediction among the feature values.

Let N be the number of risk factors, and K be the number of machine learning models. The algorithm proposed that employs the *t*-test has a time complexity of O(N^2^logN + N × K) and that using a SHAP has a time complexity of O(K × N^2^log N), assuming a linear approximation in the Shapley evaluation method.

## 3. Results

### 3.1. Ranking

Algorithm 1 was applied to the dataset after performing SMOTE, initially using a *t*-test and then SHAP as a ranking measure. It can be inferred from Table 1 that risk factors, such as glycosylated hemoglobin and systolic blood pressure, were found to be the top three risk factors for diabetic retinopathy in all machine learning (ML) models when using a *t*-test for ranking. Furthermore, it can be inferred from Table 2 that risk factors, such as systolic blood pressure and history of hypertension, were found to be among the top five risk factors for diabetic retinopathy in all machine learning models using SHAP for ranking. Table 3 shows that when an ensemble of five models were used, risk factors, such as systolic blood pressure and duration of diabetes mellitus, were found to be in the top four risk factors for DR in both the *t*-test and SHAP-based rankings.

### 3.2. Classification Performance

**Figure 2:**
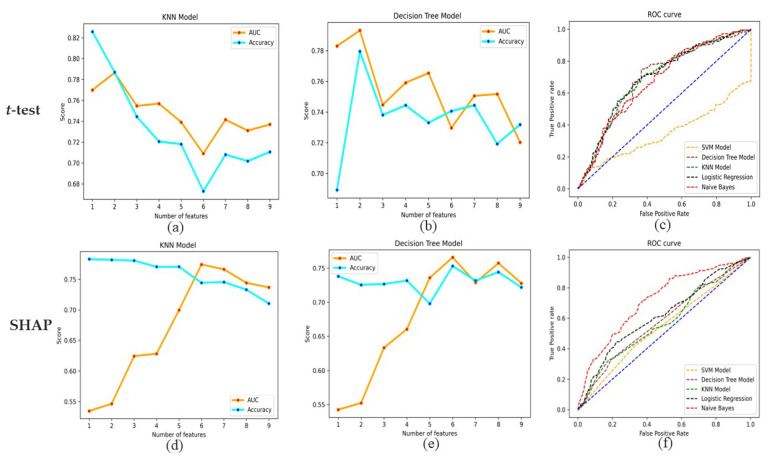
(**a**,**b**,**d**,**e**) AUC and accuracy for the Decision tree and K-Nearest Neighbors in the case of *t*-test and SHAP. (**c**,**f**) ROC curve for all models of *t*-test and SHAP.

Table 4 and Table 5 show the results for the sensitivity, specificity, AUC, and accuracy of the five individual classifiers using ensemble weights for DR prediction in the *t*-test and SHAP. All the models achieved sensitivity ranging from 0.55 to 0.76, specificity ranging from 0.59 to 0.84, AUCs ranging from 0.71 to 0.79, and accuracy ranging from 64.3% to 82.6%. Out of the five machine learning classifiers, in terms of sensitivity, Naive Bayes performed the best, with a value of 0.76 in the *t*-test and SHAP. Regarding specificity, KNN performed the best, with a value of 0.84 in the *t*-test and 0.8 in SHAP. In terms of AUC, while using a *t*-test, DT and KNN resulted in the highest AUC value of 0.79 with associated risk factors, such as glycosylated hemoglobin and systolic blood pressure. Similarly, with SHAP, DT and KNN resulted in the highest AUC of 0.77 with associated risk factors, such as systolic blood pressure, history of hypertension, diabetes mellitus duration, insulin treatment, fasting plasma glucose, and glycosylated hemoglobin. KNN achieved the best accuracy of 82.6% in the case of the *t*-test and 78.3% in the case of SHAP. The AUC and accuracy of the DT and KNN of the *t*-test and SHAP are shown in Figure 2a,b,d,e. The receiver operating characteristic (ROC) curve for all models of the *t*-test and SHAP is shown in Figure 2c,f.

## 4. Discussion

In this study, we propose an algorithm for ranking risk factors to predict DR. Systolic blood pressure was consistently found to be among the top risk factors using the *t*-test, SHAP, and ensemble methods. The sensitivity, specificity, and AUC values for the *t*-test and SHAP are very close to each other, which validates our two methods (using the *t*-test and SHAP) for ranking risk factors. In the case of the *t*-test, DT and KNN resulted in the highest AUC value of 0.79 with associated risk factors, such as glycosylated hemoglobin and systolic blood pressure. Similarly, with SHAP, DT and KNN resulted in the highest AUC value of 0.77 with associated risk factors, such as systolic blood pressure, history of hypertension, diabetes mellitus duration, insulin treatment, fasting plasma glucose, and glycosylated hemoglobin. Comparing the risk factors for AUC, it can be inferred that systolic blood pressure and glycosylated hemoglobin seem to be the critical risk factors for predicting DR.

This study uses two techniques to rank the risk factors: the *t*-test and SHAP. SHAP and *t*-tests take fundamentally different approaches to evaluate the significance of a feature. SHAP values are derived from cooperative game theory and evaluate the contribution of each feature to a specific instance’s prediction. In contrast, *t*-tests are a statistical method for testing hypotheses that compares the means of two groups. These distinct methodologies can result in varying conclusions regarding the significance of a feature. SHAP values can detect complex interactions between features that a *t*-test might overlook. In isolation, the *t*-test may indicate that HbA1c is significant, but in the context of other features and their interactions, SHAP values may indicate that HbA1c is not as significant. This may result from confounding variables, multicollinearity, or other intricate feature interactions. A *t*-test’s results can be sensitive to sample size and variation. The *t*-test might mistakenly identify HbA1c as a significant factor when it is not due to a lack of statistical power resulting from a small sample size or high variability. In contrast, SHAP values may be more resilient in such circumstances. While age is consistently at the bottom, systolic blood pressure and diabetes mellitus duration are consistently at the top of the ranking in all the models using the *t*-test and SHAP.

We can infer from Table 1, Table 2 and Table 3 that age is the least significant risk factor as it was ranked among the last five risk factors by all machine learning models and the ensemble model. The current literature shows the effect of age on the severity of DR is unclear and varies with the population being studied [36]. While Stratton et al. reported that old age impacts the progression of DR [50], other studies identified younger age as a risk factor [51,52,53]. Therefore, it is likely that the risk of DR may be present irrespective of age; hence, screening should be performed in all age groups.

Although age was not on the list of the top risk factors in this analysis, we did find that duration of diabetes was one of the top four risk factors employing ensemble models of five classifiers. The duration of diabetes is related to the patient’s exposure time to other DR-related risk factors. It should, therefore, be of prime importance while targeting screening towards DR. Previous studies have shown that DM duration may be the most important independent risk factor for DR [54,55,56].

There were few investigations on the risk stratification of DR based on ML and risk factors. Azizi-Soleiman et al. [57] reported a model for detecting DR in Iranians using outpatient clinical data. The logit model obtained an AUC of 0.760 by training on the data of 1782 patients (without cross-validation) using backward elimination as a feature selection strategy. Tsao et al. [33] divided the clinical data of 536 Taiwanese patients into training and validation sets (80:20 ratio) and tested how well the four models (Support Vector Machine, Decision Tree, Artificial Neural Network (ANN), and Logistic Regression) could detect DR. They found that the Support Vector Machine performed the best, with an AUC of 0.839. According to Yao et al. [58], an Artificial Neural Network with back propagation outperformed Logistic Regression in DR detection with AUCs of 0.84 and 0.77, respectively. Population-based data are more pertinent to the reality of DR screening programs than hospital-based data [59]. Our study applied machine learning (ML) techniques to population-based data and demonstrated their utility for DR detection. Moreover, we have proposed two techniques to rank the risk factors: the *t*-test and SHAP, which validate each other, obtaining AUCs of 0.79 and 0.77, respectively.

The first limitation of the study is that only a subset of the risk factors suggested by the current literature were considered. There is scope for a larger set of risk factors to be considered to identify the top risk factors that can aid in initial screening for referable DR in populations where ophthalmologists are scarce. Second, it was not possible to evaluate risk factor rankings for each form of retinopathy separately in the present study. The classification of risk factors refers to the risk of diabetic retinopathy, regardless of its severity.

Our study has shown that ML technology successfully ranks important risk factors in large-scale epidemiological studies. Previous studies have demonstrated the vital role of ML in other medical fields, such as T2DM, obesity, and heart failure [60,61,62]. Our results confirm the excellent performance of ML in predicting diabetic retinopathy. This is the first study that evaluates the importance of risk factors using various ML methods with data from the Indian population and checks the risk factors for diabetic retinopathy.

## 5. Conclusions

The study aims to reflect the importance of ranking risk factors to find their relevance to DR. We have proposed two techniques to find the relative contributions of risk factors to the presence of DR. In both cases, age contributed the least and systolic blood pressure contributed the most among the nine risk factors considered for the study. Thus, validating both of our proposed techniques, KNN achieved the best accuracy of 82.6% in the case of the *t*-test and 78.3% in the case of SHAP to predict DR. A subset of risk factors given by ophthalmologists was considered in the study. Furthermore, other risk factors, such as demographics, lifestyle, family history, living standards, and ethnicity, need to be explored in further studies as part of the future scope of this work. These risk factors could aid in developing a risk factor algorithm for DR and aid in the prescreening of DR. The algorithm can be a prescreening tool using fundus photographs to identify referable and non-referable DR. The study can also be extended using images and top risk factors to predict DR. Moreover, the ranking of risk factors for non-proliferative/proliferative DR or diabetic macular edema and whether the ranking would change with the development of macular edema or proliferative DR could be a potential future study.

## Figures and Tables

**Table 1:** Ranking of risk factors using *t*-test and SMOTE with various ML models.

SNo.	Decision Tree	SVM	KNN	Logistic Regression	Naive Bayes
1	glycosylated hemoglobin	glycosylated hemoglobin	glycosylated hemoglobin	glycosylated hemoglobin	glycosylated hemoglobin
2	diabetes mellitus duration	systolic blood pressure	systolic blood pressure	body mass index	body mass index
3	systolic blood pressure	diabetes mellitus duration	fasting plasma glucose	systolic blood pressure	systolic blood pressure
4	fasting plasma glucose	insulin treatment	history of hypertension	gender	gender
5	history of hypertension	fasting plasma glucose	insulin treatment	age	diabetes mellitus duration
6	insulin treatment	history of hypertension	diabetes mellitus duration	insulin treatment	age
7	gender	gender	gender	fasting plasma glucose	insulin treatment
8	body mass index	body mass index	body mass index	history of hypertension	fasting plasma glucose
9	age	age	age	diabetes mellitus duration	history of hypertension

**Table 2:** Ranking of risk factors using SHAP and SMOTE with various ML models.

SNo.	Decision Tree	SVM	KNN	Logistic Regression	Naive Bayes
1	diabetes mellitus duration	systolic blood pressure	glycosylated hemoglobin	systolic blood pressure	systolic blood pressure
2	glycosylated hemoglobin	fasting plasma glucose	systolic blood pressure	history of hypertension	fasting plasma glucose
3	systolic blood pressure	history of hypertension	fasting plasma glucose	insulin treatment	history of hypertension
4	fasting plasma glucose	insulin treatment	history of hypertension	diabetes mellitus duration	insulin treatment
5	history of hypertension	diabetes mellitus duration	insulin treatment	gender	diabetes mellitus duration
6	insulin treatment	gender	diabetes mellitus duration	glycosylated hemoglobin	gender
7	gender	glycosylated hemoglobin	gender	body mass index	glycosylated hemoglobin
8	body mass index	body mass index	body mass index	fasting plasma glucose	body mass index
9	age	age	age	age	age

**Table 3:** Ranking of risk factors using an ensemble of ML models.

SNo.	*t*-test + Ensemble	Shapely + Ensemble
1	glycosylated hemoglobin	systolic blood pressure
2	systolic blood pressure	history of hypertension
3	body mass index	diabetes mellitus duration
4	diabetes mellitus duration	insulin treatment
5	gender	fasting plasma glucose
6	age	glycosylated hemoglobin
7	insulin treatment	gender
8	fasting plasma glucose	body mass index
9	history of hypertension	age

**Table 4:** Metrics for ranking using a *t*-test using ensemble weights (Support Vector Machines (SVM), K-Nearest Neighbors (KNN), and Area under the ROC Curve (AUC)).

*t*-test				
Model	Sensitivity	Specificity	AUC	Accuracy
Decision Tree	0.6	0.83	0.79	0.779
SVM	0.72	0.66	0.75	0.711
KNN	0.58	0.84	0.79	0.826
Logistic Regression	0.68	0.65	0.71	0.657
Naive Bayes	0.76	0.59	0.73	0.668

**Table 5:** Metrics for ranking using Shapely additive explanations (SHAP) using ensemble weights (Support Vector Machines (SVM), K-Nearest Neighbors (KNN), and Area under the ROC Curve (AUC)).

SHAP				
Model	Sensitivity	Specificity	AUC	Accuracy
Decision Tree	0.64	0.78	0.77	0.753
SVM	0.72	0.66	0.75	0.747
KNN	0.55	0.8	0.77	0.783
Logistic Regression	0.68	0.65	0.71	0.66
Naive Bayes	0.76	0.59	0.73	0.643

## Data Availability

The datasets analyzed during the current study are not publicly available as it is against the organization/hospital policy. However, they are available from the corresponding author on reasonable request.
